# Healthcare use and clinical investigations before a diagnosis of ovarian cancer: a register-based study in Denmark

**DOI:** 10.1186/s12875-023-02132-3

**Published:** 2023-08-30

**Authors:** Isabella Gringer Rousing, Peter Vedsted, Peter Hjertholm, Per Kallestrup, Marie-Louise Ladegaard Baun, Line Flytkjaer Virgilsen

**Affiliations:** 1 Research Unit for General Practice, Bartholins Allé 2, Aarhus, 8000 Denmark; 2 Department of Public Health, Aarhus University, Bartholins Allé 2, Aarhus, 8000 Denmark; 3 University Clinic for Innovative Patient Pathways, Diagnostic Center, Silkeborg Regional Hospital, Department of Clinical Medicine, Aarhus University, Falkevej 3, Silkeborg, 8600 Denmark

**Keywords:** General practice, Diagnosis, Ovarian neoplasms, Universal health care, Registries, Denmark

## Abstract

**Background:**

Ovarian cancer (OC) is associated with a poor prognosis, which calls for earlier diagnosis. This study aimed to analyse the health care use in primary care and at hospitals among women with OC compared to non-cancerous women to identify a window of opportunity for earlier diagnosis.

**Methods:**

This nationwide register-based observational cohort study included all Danish women aged ≥ 40 years who were diagnosed with a first-time OC or borderline ovarian tumour in 2012–2018 and with no previous cancer diagnosis (n = 4,255). For each case, ten non-cancerous women were identified (n = 42,550). We estimated monthly incidence rate ratios using a negative binomial regression model to assess the use of health care services. We calculated risk ratios of having multiple contacts to general practice before a diagnosis using a binary regression model.

**Results:**

Cases had statistically significantly higher contact rates to general practice from five months prior to the diagnosis compared to references. From six to eight months prior to diagnosis, an increased use of transvaginal ultrasound and gynaecologist was seen for cases compared to references.

**Conclusions:**

Increased healthcare use was seen relatively closely to the time of diagnosis for women with OC. This indicates a narrow window of opportunity for a timelier diagnosis. Still, the use of specialised assessment increased at six to eight months before the diagnosis. When women present unspecific symptoms, awareness of potential ovarian malignancies and safety-netting by the general practitioner may be pivotal.

**Trial registration:**

Not relevant.

**Supplementary Information:**

The online version contains supplementary material available at 10.1186/s12875-023-02132-3.

## Background

Ovarian cancer (OC) has an age-standardised annual incidence rate of 16.1 per 100,000 women in Denmark [1]. Women with OC (including ovarian, tubal, and primary peritoneal cancer) have a poor prognosis, which is likely to be due to advanced disease at the time of diagnosis [2, 3]. Identifying patients with early-stage disease is important as early diagnosis may lead to improved prognosis [2].

As screening for OC is not available or recommended [4], the key to ensuring early diagnosis is referral from the general practitioner (GP) to diagnostic investigation [5]. Recent studies have shown that nine in ten OC patients consulted their GP with symptoms in the year prior to diagnosis [6, 7].

Yet, OC is known to present with vague and unspecific symptoms, such as abdominal pain or bloating, constipation, or increased urination frequency [8]. Among women consulting their GP prior to an OC diagnosis six in ten presented vague and unspecific symptoms, and they were less likely to be referred to a cancer patient pathway (CPP) compared to women with suspected cancer [6]. When symptoms mirror benign disease and CPP referral is not considered, the diagnostic interval is generally prolonged [6]. If the patient is not referred to a CPP, the GP may refer the patient to another specialist (e.g. gynaecologist or urologist), depending on the nature of symptoms, and this is often associated with prolonged diagnostic interval [9]. Thus, the complex symptomatology and the low incidence of OC challenge the diagnosis of OC in general practice.

Danish studies have shown increased diagnostic activity several months prior to diagnosis for other cancer types [10, 11]. However, we do not know whether such a window exists for patients with OC.

## Methods

### Aim

We aimed to analyse the health care use in primary care and at hospitals, including diagnostic investigations, among women with OC compared to non-cancerous women.

### Study design

This observational cohort study was based on register-based data linked at the individual level by the unique civil registration number, which is assigned to all Danish citizens at birth or immigration [12].

### Setting

More than 98% of all citizens are listed with a general practice in Denmark [13]. The Danish healthcare system is tax-funded and offers free access to healthcare services for residents. GPs and private medical specialists are self-employed but working on contract with the public funding authorities [13]. The GP acts as a gatekeeper to secondary care; if contact to other specialist (private or public) is needed, it requires a referral from the patients GP, except hospital emergency services, ear-nose-throat and eye specialists who can be accessed directly [13]. Before a referral, a physical examination should be performed, i.e. an gynaecological examination when referred to a gynaecologist. The GP has access to a wide range of laboratory tests including point-of-care tests (POCT), while diagnostic imaging and invasive procedures are done by referral to specialists. When a GP suspects cancer, the GP should refer the patient directly to a CPP to ensure fast diagnosis [14]. The CPP includes contact to relevant specialists for the suspected cancer type, i.e. the patient is seen by a gynaecologist if the GP suspect a gynaecological cancer. The implementation of CPPs for 32 cancer types began in Denmark in 2007, and the CPP for OC was implemented in 2009 [5, 14].

### Study population

The study population consisted of all women registered with a first-time OC or borderline ovarian tumour (BOT) according to the International Classification of Diseases, 10th revision (ICD-10 C48, C56, C57 and D39.1) in the Danish Cancer Registry (DCR) [15] or in the Danish Gynecological Cancer Database (DGCD) [16] from 1 January 2012 to 31 December 2018 and aged ≥ 40 years at the time of diagnosis (n = 4,255).

**Fig. 1:**
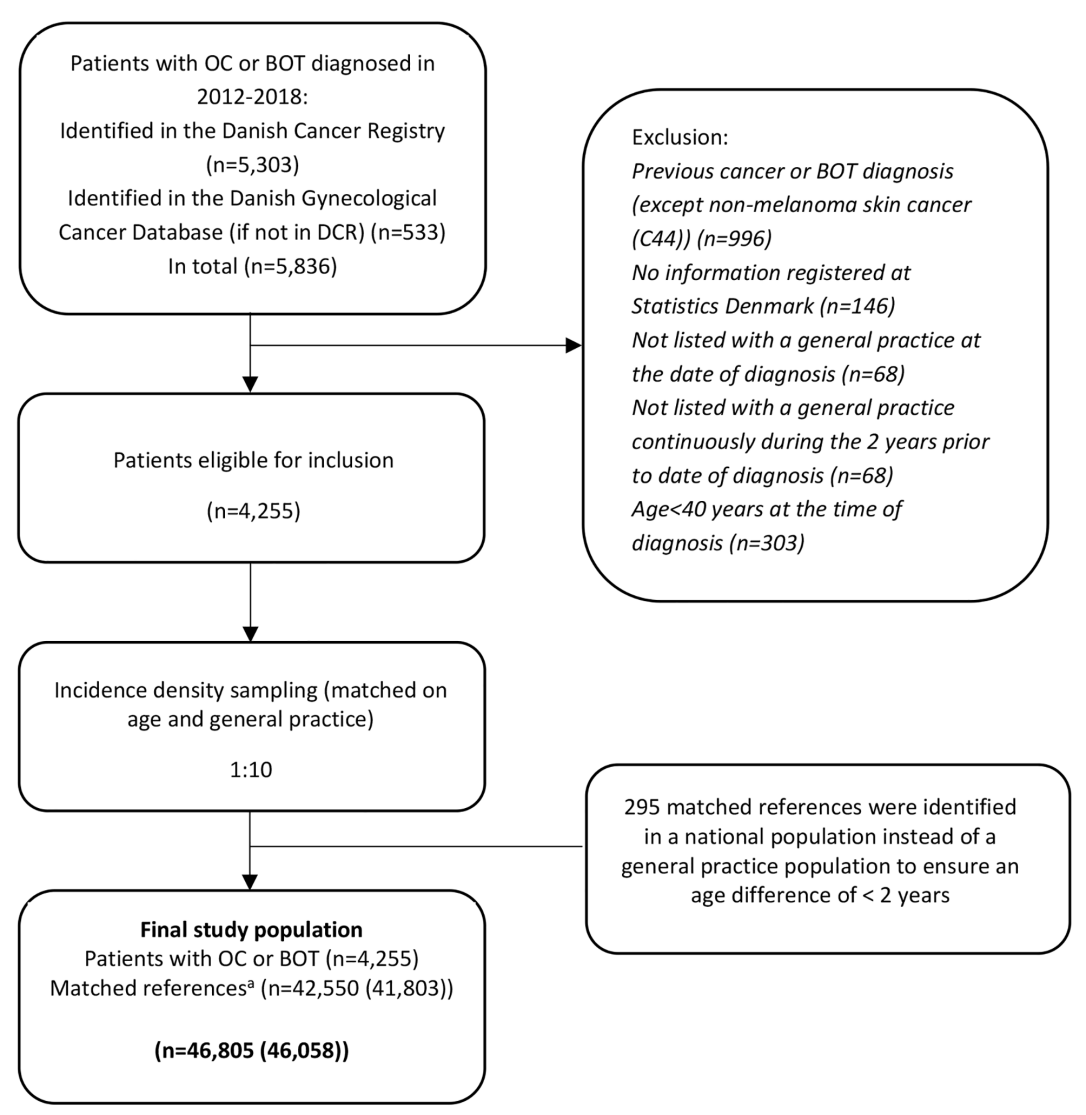
Flowchart of inclusion of study population ^a^The total number of observations is listed. Matched references may serve as comparison subjects to more than one case. Unique numbers of women are in brackets Abbreviations: BOT = borderline ovarian tumour; OC = ovarian cancer

For each case, we used incidence density sampling to identify ten non-cancerous women matched on age and general practice (n = 42,550), see Fig. 1. References were identified in the Civil Registration System [12]. A reference could be included as a case after the index date in accordance with the density sampling method [17]. One matched reference could serve as a comparison subject for more than one case. An index date was assigned to all participants. For women with OC or BOT, the index date was defined as the date of diagnosis. For the matched population, the index date was defined as the date of diagnosis for the corresponding case. In case of mismatch between the DCR and the DGCD, the DCR was considered the primary registry. Women with a previous cancer or BOT diagnosis were excluded, except from non-melanoma skin cancer (C44). To ensure a stable GP affiliation, both groups were required to live in Denmark and to have been affiliated with a general practice during the 24 months prior to the index date (Fig. 1). Both cases and controls were allowed to change their general practice.

### Outcomes and data

Main outcomes were based on data from general practice, specialised services in primary care, and services in secondary care, including public and private hospitals. All outcomes were assessed monthly from 12 months prior to the index date. Data from primary care was obtained from the Danish National Health Insurance Service Register [18] and included daytime consultations (face-to-face and home visits), haemoglobin (Hb) measurements (POCT), urine dipstick tests (POCT) and blood tests in general practice, contacts to private practicing gynaecologists, and diagnostic investigations. The data used to estimate outcomes in secondary care was obtained from the Danish National Patient Register (DNPR) [19] and included contacts to a department of gynaecology or urology, diagnostic investigations performed at a hospital, and CPP referrals (excluding the CPP for OC). We included data on CPPs from 2014, as CPP registrations were not mandatory in the DNPR until 1 October 2012 [19]. Diagnostic investigations included colonoscopies (including sigmoidoscopies), transvaginal ultrasound (TVUS), abdominal ultrasound, and computed tomography (CT) performed by practicing medical specialists or at a hospital. A full list of procedure codes is presented in Appendix A.

### Covariates

The following variables were included to adjust for differences between groups. Statistics Denmark provided data on educational level, marital status, income and country of origin [20]. Educational level was defined as the highest obtained level of education at study entry (at 12 months prior to index date) and divided into three groups (Short, Medium, Long) according to the International Standard Classification of Education 2011 [21]. Missing educational level (n = 888) was recoded as “Short” in line with previous research [6]. Marital status was divided into two groups (Married/cohabitating, Single) based on the year of study entry. Information on income was obtained 12 months prior to the index date to account for income loss due to illness absence and was divided into tertiles (Low, Middle, High) based on the adjusted household income by the Organisation for Economic Co-operation and Development [22]. Country of origin was divided into two groups (Danish/descendant, Immigrant).

The Charlson’s Comorbidity Index (CCI) was used to estimate comorbidity following the method by Quan [23]. The CCI score (excluding cancer) was based on diagnosis registrations in the DNPR at 10 years preceding the first day of analysis (i.e. the first date in the first month included in the analysis of activities performed in general practice and at hospitals) and divided into three groups (None: score of 0, Low: score of 1–2, High: score of > 2). For cases, information on tumour stage registered according to the International Federation of Gynecology and Obstetrics (FIGO) was obtained from the DGCD. In case of more than one registration at index date, the highest tumour grade was chosen. If not available from the DGCD (n = 511), FIGO stage was calculated based on tumour-stage information from the DCR [24].

### Statistical analysis

We used a negative binomial regression model applying cluster robust variance estimation at the patient level to calculate incidence rate ratios (IRR) to compare the monthly healthcare use rates between cases and references in the year prior to index date. We used a binary regression model applying cluster robust variance at the patient level to calculate the risk ratios (RR) of having more face-to-face consultations, Hb measurements, urine dipstick tests, or blood tests than the reference group within the year prior to diagnosis, while omitting the last month before diagnosis due to expected high healthcare use in this period. Risk ratios were stratified into two equal time periods based on an explorative approach. Age at index date was modelled through restricted cubic splines with four knots according to Harrell’s recommended percentiles [25]. All analyses were made as crude analyses and adjusted for all covariates.

Crude rates of contacts were displayed as histograms with 95% confidence intervals (CI). Scatter plots were used to display adjusted IRRs with 95% CIs. For graphical purposes, we excluded the last month before diagnosis in the presentation of IRRs (presented in Appendix B). All IRR estimates are presented in Appendix C.

All analyses were conducted with Stata statistical software, release 17.

## Results

The study included 4,255 patients with incident OC or BOT and 42,550 references from 1,729 general practices. Of the 4,255 patients, 21.5% had a BOT diagnosis (n = 914). Median age at index date was 66 years (interquartile range (IQR) 57;74). Sociodemographic variables and comorbidity were comparable between patients and references (Table 1).

**Table 1:** Characteristics of the study population

	Ovarian cancer or borderline ovarian tumour		No cancer		Total	
	Median	(IQR)	Median	(IQR)	Median	(IQR)
**Age at index date**	66	(57; 74)	66	(57; 74)	66	(57; 74)
	**N**	**(%)**	**N**	**(%)**	**N**	**(%)**
**Total**	4,255	(100.0)	42,550	(100.0)	46,805	(100.0)
**Sex**						
Female	4,255	(100.0)	42,550	(100.0)	46,805	(100.0)
**Age groups**^**a**^, years						
40–49	500	(11.8)	4,995	(11.7)	5,495	(11.7)
50–64	1,384	(32.5)	13,835	(32.5)	15,219	(32.5)
65–74	1,344	(31.6)	13,445	(31.6)	14,789	(31.6)
75–84	785	(18.4)	7,891	(18.5)	8,676	(18.5)
85+	242	(5.7)	2,384	(5.6)	2,626	(5.6)
**Comorbidity** ^**b**^						
None	3,346	(78.6)	32,539	(76.5)	35,885	(76.7)
Low	782	(18.4)	8,495	(20.0)	9,277	(19.8)
High	127	(3.0)	1,516	(3.6)	1,643	(3.5)
**Educational level** ^**c**^						
Short	1,551	(36.5)	16,052	(37.7)	17,603	(37.6)
Medium	1,687	(39.6)	16,895	(39.7)	18,582	(39.7)
Long	1,017	(23.9)	9,603	(22.6)	10,620	(22.7)
**Disposable income** ^**d**^						
Low	1,362	(32.0)	14,084	(33.1)	15,446	(33.0)
Middle	1,457	(34.2)	13,988	(32.9)	15,445	(33.0)
High	1,436	(33.7)	14,478	(34.0)	15,914	(34.0)
**Marital status** ^**d**^						
Cohabitant	2,301	(54.1)	23,877	(56.1)	26,178	(55.9)
Living alone	1,954	(45.9)	18,673	(43.9)	20,627	(44.1)
**Country of origin**						
Danish	4,029	(94.7)	39,827	(93.6)	43,856	(93.7)
Immigrant	226	(5.3)	2,723	(6.4)	2,949	(6.3)
**Tumour stage** ^**e**^						
I	1,416	(33.3)	n/a	n/a	1,416	(3.0)
II	245	(5.8)	n/a	n/a	245	(0.5)
III	1,245	(29.3)	n/a	n/a	1,245	(2.7)
IV	972	(22.8)	n/a	n/a	972	(2.1)
Unknown	377	(8.9)	n/a	n/a	377	(0.8)
Reference	n/a	n/a	42,550	(100.0)	42,550	(90.9)

^a^Age at the time of diagnosis, i.e. index date

^b^Charlson Comorbidity Index score calculated on the first day of analysis, divided into 0 (none) 1–2 (low) and ≥ 3 (high)

^c^Educational level defined as the highest completed level of education at study entry (12 months prior to index date)

^d^Disposable income and marital status defined at study entry (12 months prior to index date)

^e^Tumour stage according to FIGO classification. Borderline tumours included

Abbreviations: n/a = Not applicable. IQR = Interquartile range

### General practice

**Fig. 2:**
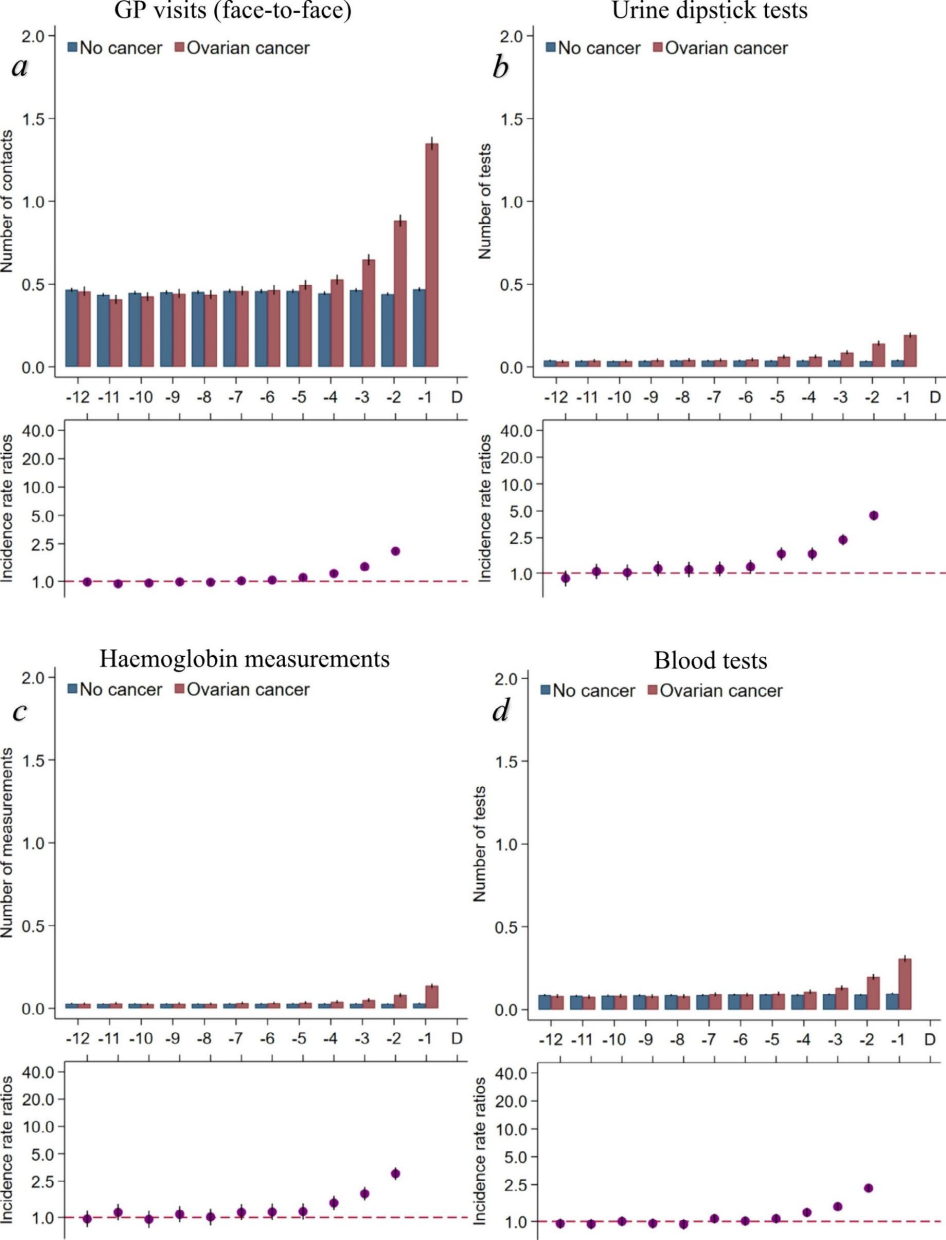
Consultation rates and tests in general practice in the year preceding an ovarian cancer diagnosis (omitting the IRR in the last month before diagnosis) Number of contacts or tests in general practice stratified on ovarian cancer (yes/no). Borderline ovarian tumours included. Number of contacts/tests are presented as crude rates of mean number of contacts/tests per month. Incidence rate ratios were adjusted for age, comorbidity, educational level, marital status, disposable income and country of origin. Black lines represent 95% confidence intervals Abbreviations: IRR = Incidence rate ratio

In the year prior to the index date (omitting the last month before diagnosis), 91.0% of cases and 86.4% of references had at least one face-to-face consultation in general practice. The rates of face-to-face consultations and urine dipstick tests were statistically significantly higher from five months prior to diagnosis for cases compared to references (Fig. 2a and b). The use of Hb measurements and blood tests was statistically significantly higher from four months prior to diagnosis for cases compared to references (Fig. 2c and d).

No difference was observed between the two groups in the risk of having more contacts to general practice from month − 12 to month − 6.5. However, we found a higher risk among cases of having more urine dipstick tests performed (RR = 1.09 (95% CI 1.01–1.18)) (Table 2).

**Table 2:** Proportions of women with face-to-face consultations, point-of-care haemoglobin measurements, urine dipstick tests and blood tests in general practice during the year prior to the index date (omitting the last month before index date), including risk ratios. Stratified on number of months prior to index date

	Month − 12 to -6.5						Month − 6.5 to -2					
	Cases		References				Cases		References			
	n	(%)	n	(%)	RR	95% CI*	n	(%)	n	(%)	RR	95% CI*
**All**	4,255	(100)	42,550	(100)			4,255	(100)	42,550	(100)		
**Consultations**												
0	1,223	(28.74)	11,856	(27.86)	1.02	0.97–1.07	712	(16.73)	11,649	(27.38)	**0.60**	**0.56–0.65**
1–4	2,304	(54.15)	23,292	(54.74)	0.99	0.96–1.02	2,416	(56.78)	23,528	(55.29)	1.03	1.00-1.06
5–8	548	(12.88)	5,528	(12.99)	1.01	0.93–1.10	853	(20.05)	5,540	(13.02)	**1.56**	**1.46–1.66**
≥9	180	(4.23)	1,874	(4.40)	1.01	0.87–1.17	274	(6.44)	1,833	(4.31)	**1.56**	**1.38–1.76**
**Hb measurements**												
0	3,766	(88.51)	37,641	(88.46)	1.00	0.99–1.01	3,495	(82.14)	37,667	(88.52)	**0.93**	**0.92–0.94**
≥1	489	(11.49)	4,909	(11.54)	1.01	0.94–1.10	760	(17.86)	4,883	(11.48)	**1.57**	**1.48–1.68**
**Urine dipstick tests**												
0	3,686	(86.63)	37,227	(87.49)	0.99	0.98-1.00	3,164	(74.36)	37,114	(87.22)	**0.85**	**0.84–0.87**
≥1	569	(13.37)	5,323	(12.51)	**1.09**	**1.01–1.18**	1,091	(25.64)	5,436	(12.78)	**1.99**	**1.88–2.10**
**Blood tests**												
0	3,022	(71.02)	30,070	(70.67)	1.00	0.99–1.02	2,523	(59.29)	29,433	(69.17)	**0.86**	**0.84–0.88**
≥1	1,233	(28.98)	12,480	(29.33)	1.01	0.96–1.05	1,732	(40.71)	13,117	(30.83)	**1.31**	**1.26–1.36**

Abbreviations: RR = risk ratio; CI = confidence interval; Hb = Haemoglobin

*Adjusted analyses comparing the activity between cases and references in general practice. RRs adjusted for age, disposable income, educational level, marital status, country of origin and comorbidity according to the Charlson Comorbidity Index. Significant findings in bold

### Private practicing medical specialists and hospital

**Fig. 3:**
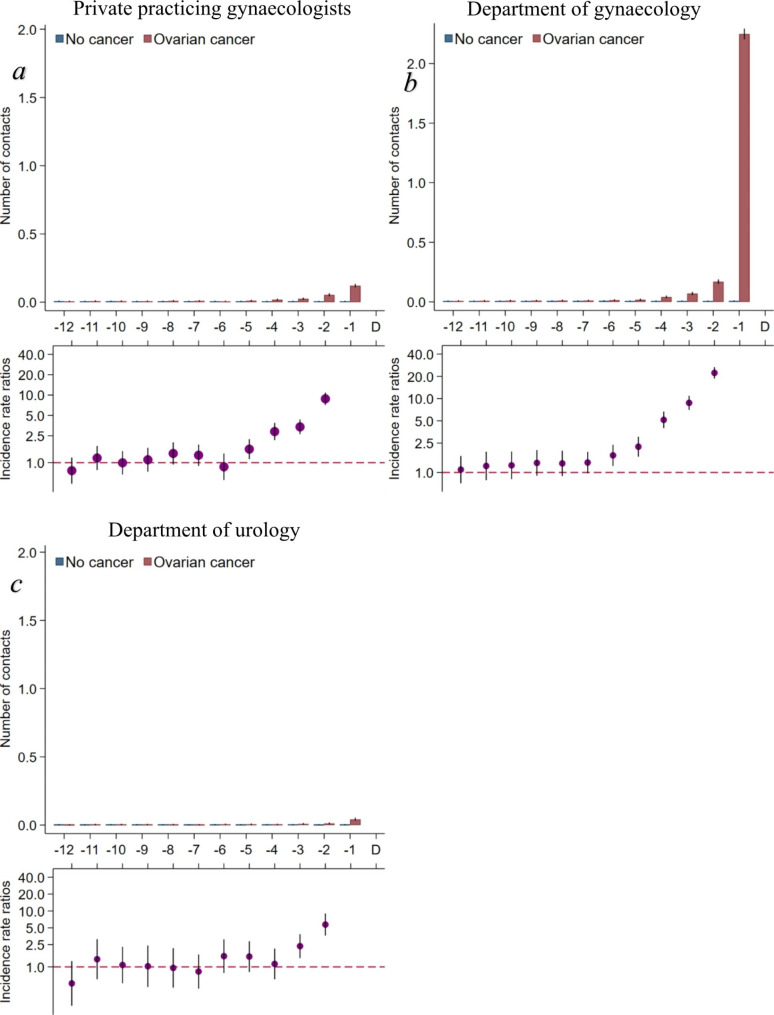
Contacts to relevant specialists in the year preceding an ovarian cancer diagnosis (omitting the IRR in the last month before diagnosis) Number of consultations in different healthcare departments stratified on ovarian cancer (yes/no). Borderline ovarian tumours included. Maximum one contact at each department/private specialist per women per day included. Number of consultations are presented as crude rates of mean number of consultations per month. Incidence rate ratios were adjusted for age, comorbidity, educational level, marital status, disposable income and country of origin. Black lines represent 95% confidence intervals Abbreviations: IRR = Incidence rate ratio

**Fig. 4:**
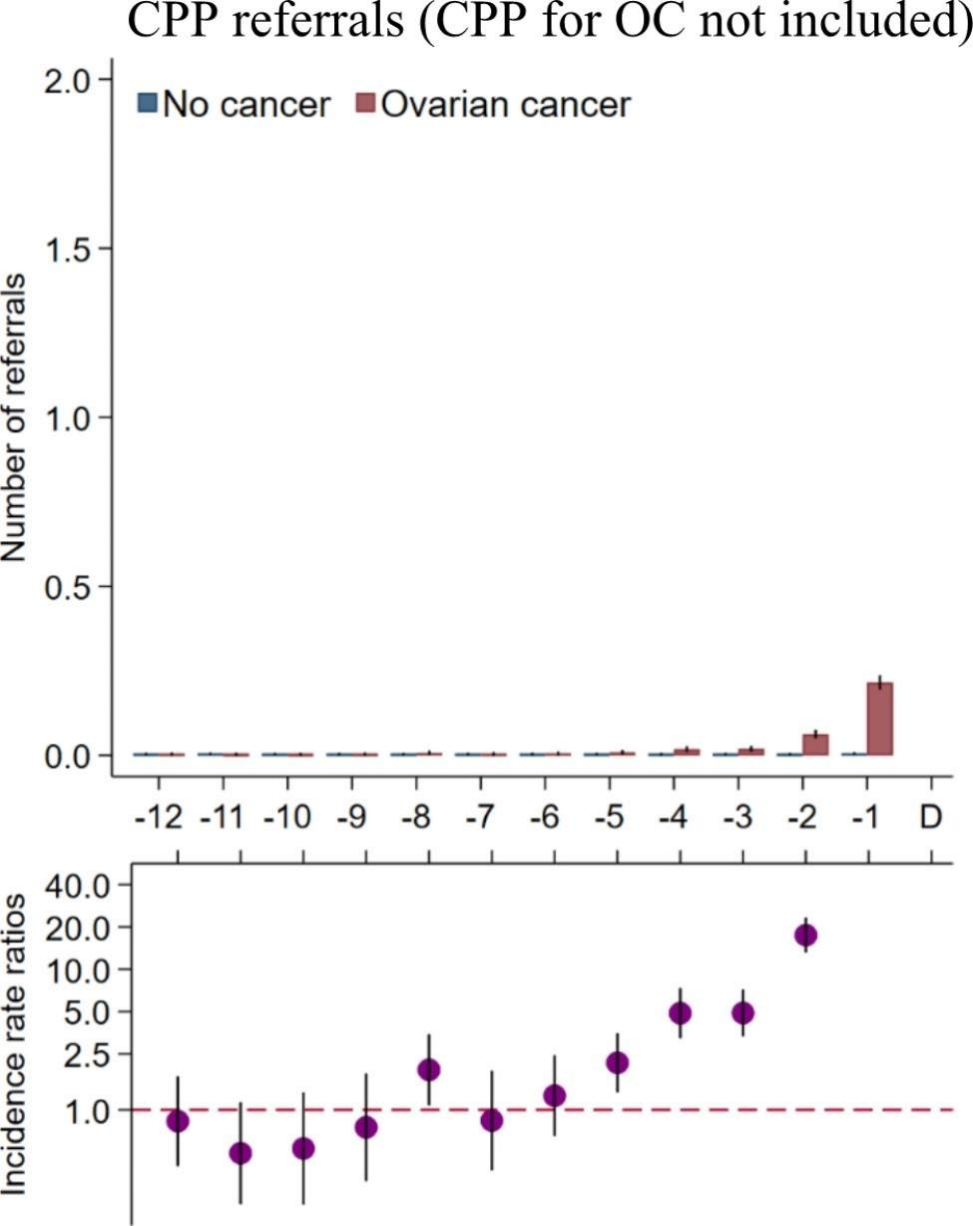
Cancer patient pathway (CPP) referrals in the year preceding an ovarian cancer diagnosis (omitting the IRR in the last month before diagnosis). The CPP for OC was not included Number of CPP referrals in 2014–2018 stratified on ovarian cancer (yes/no). Borderline ovarian tumours included. Maximum one CPP referral per women per day included. CPP for OC not included. Number of investigations are presented as crude rates of mean number of CPPs per month. Incidence rate ratios were adjusted for age, comorbidity, educational level, marital status, disposable income and country of origin. Black lines represent 95% confidence intervals. Abbreviations: CPP = cancer patient pathway; IRR = Incidence rate ratio; OC = ovarian cancer.

The contact rates to gynaecologists increased statistically significantly from five to six months prior to diagnosis for cases compared to references (Fig. 3a-b). The CPP referral rates (excluding the CPP for OC) were statistically significantly higher from five months prior to diagnosis (Fig. 4), and the contact rates to a department of urology were statistically significantly higher from three months prior to diagnosis for cases compared to references (Fig. 3c).

### Diagnostic investigations

**Fig. 5:**
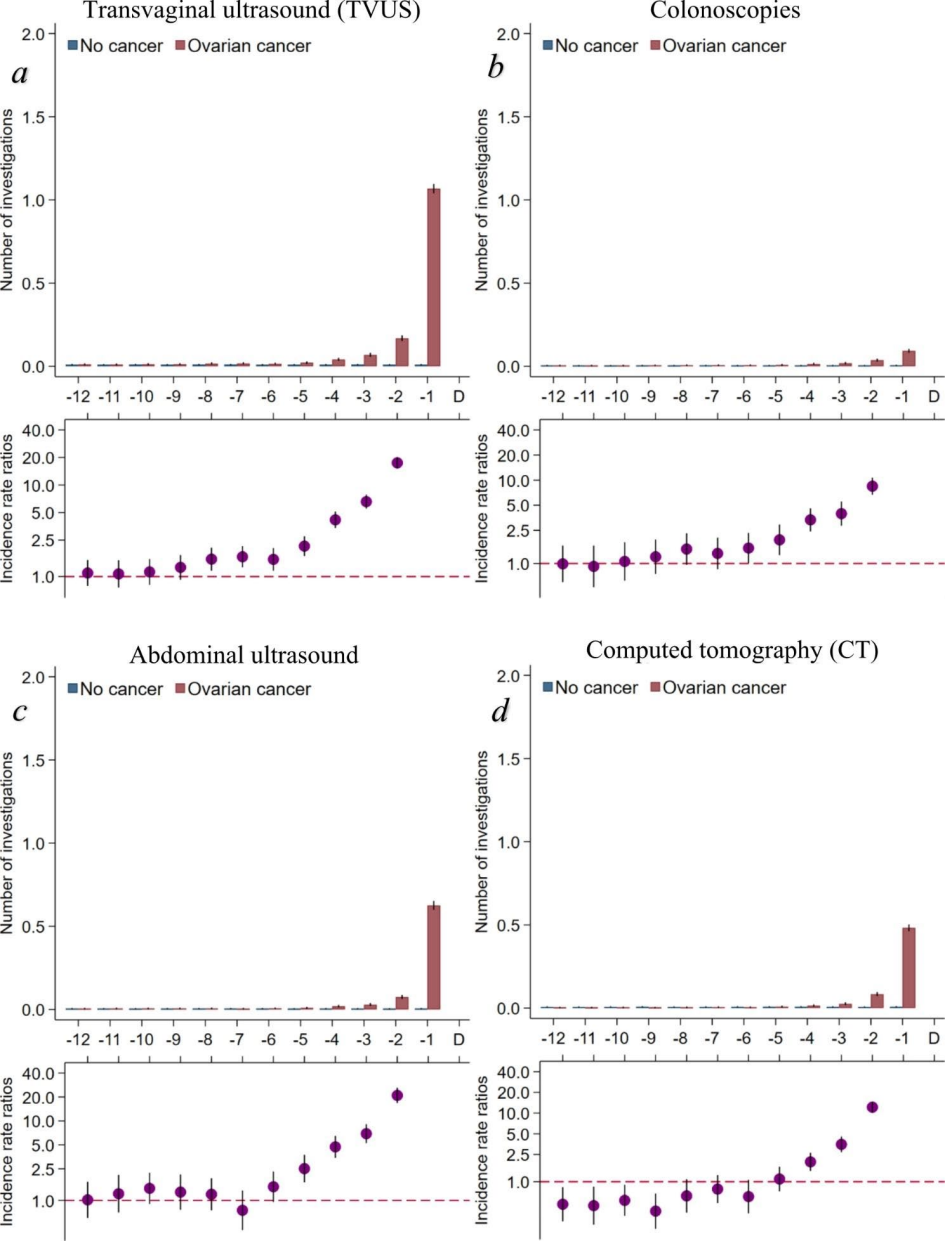
Diagnostic investigations made by private and hospital specialists in the year preceding an ovarian cancer diagnosis (omitting the IRR in the last month before diagnosis) Number of different diagnostic investigations performed and stratified on ovarian cancer (yes/no). Borderline ovarian tumours included. Maximum one investigation of each type per women per day included. Number of investigations are presented as crude rates of mean number of investigations per month. Incidence rate ratios were adjusted for age, comorbidity, educational level, marital status, disposable income and country of origin. Black lines represent 95% confidence intervals. CT scans were only performed at hospitals. Abbreviations: IRR = Incidence rate ratio.

The rates of TVUS were statistically significantly higher from eight months prior to diagnosis for cases compared to references (Fig. 5a). The rates of colonoscopies were statistically significantly higher from six months prior to diagnosis for cases compared to references (Fig. 5b), and the rates of abdominal ultrasound were statistically significantly higher from five months prior to diagnosis for cases compared to references (Fig. 5c). The rates of CT scans were lower among cases compared to references until six months prior to diagnosis; from four months prior to diagnosis, the rates of CT scans were statistically significantly higher for OC patients (Fig. 5d).

Data is not shown for gastroscopies, magnetic resonance imaging, and contacts to the emergency department owing to few observations.

### Sub-analyses

When including telephone and email consultations in the monthly IRRs of face-to-face consultations, the results did not change (data not shown).

Monthly IRRs were calculated for all outcomes, excluding cases with a BOT diagnosis, and this did not change the overall findings (data not shown). Furthermore, monthly IRRs were calculated for all outcomes after stratifying patients into diagnosed before and after 2015 (due to change in CPP registration from 2014). Results from 2012 to 2014 resembled the results from 2015 to 2018 (data not shown).

## Discussion

### Main findings

From five months preceding the diagnosis, women with OC or BOT consulted their GP significantly more than the reference group, and the use of blood tests, Hb measurements, and urine dipstick tests increased simultaneously from four to five months prior to diagnosis. The use of diagnostic investigations and the number of contacts to medical specialists in secondary care increased towards the time of the diagnosis (with varying frequency and timing); the increase usually started five to six months prior to diagnosis. The use of TVUS increased significantly from eight months before diagnosis.

### Strengths and limitations

The nationwide design and the inclusion of all patients with incident OC or BOT registered in the DCR within a seven-year period were important strengths of the study. The DCR is known to have an almost complete registration of cancers diagnosed in Denmark [15], and linkage of data from the DCR to other Danish registries, which are also known as highly valid and complete, was another strength of the study [12, 18, 19]. This minimised the risk of information and selection bias. Moreover, cases and references were matched on age and general practice and were comparable regarding socioeconomic and sociodemographic factors, which reduced the risk of confounding by these variables. The study population was restricted to women with a first-time cancer or first-time BOT to avoid the influence of increased alertness from the GP towards patients with a previous history of cancer or BOT; thus, the results applied only to patients with incident cancer. Women with a BOT diagnosis were included along with women with an OC diagnosis, as these expel similar symptoms and signs. The sensitivity analysis showed comparable results after excluding women with a BOT diagnosis.

A limitation of the study was that the reasons for encounter could not be identified in the healthcare system as registry data is collected for other purposes and does not contain this information. Likewise, information on gynaecological examinations performed are not registered in national databases. Moreover, some GPs may have used blood tests instead of a POCT to measure Hb level, which might have led to misclassification in the analysis of Hb measurements. However, such misclassification would be nondifferential and may have resulted in an underestimation of the associations found for Hb measurements [26]. The Hb measurements, blood tests, and urine dipstick tests were included as indicators of relevant diagnostic investigations and considerations made in general practice, although they are not specific for cancer diagnostics.

The results are considered generalisable to other countries with similar healthcare structures, where the GP acts as a gatekeeper to the rest of the healthcare system.

### Comparison with other studies and clinical implications

To our knowledge, this is the first study to investigate the frequency and timing of healthcare use in the year preceding a diagnosis for OC patients compared to non-cancerous women. The inclusion of women with a BOT diagnosis prompted a higher percentage of women with low-stage disease (Table 1) than previously observed for OC [6]. Our findings of increased consultation frequency in general practice and higher use of diagnostic investigations in the year preceding an OC diagnosis are consistent with former studies using the same methodology for different cancer types [10, 27, 28]. However, the timing of the increase in contacts to general practice differed between different types of cancer. For intracranial cancers, the patterns were comparable to this study [28]. However, a study on colorectal cancer found increased consultation frequency in general practice from nine months prior to diagnosis and higher rates of Hb measurements (POCT) from 17 months preceding diagnosis [10]. A recent study examining the investigation rates for 11 types of abdominal cancer showed that a TVUS was often performed during the year preceding an OC diagnosis and also found increased rates of colonoscopy from six months preceding an OC diagnosis [11]. This is in line with the findings of the present study. We demonstrated that the IRR of having a TVUS performed rose from eight months prior to the OC diagnosis, which was three months earlier than the rise seen in contacts to general practice. This indicates that the GP at an earlier stage had referred the women to a gynaecologist which did not lead to an immediate diagnosis. This may be explained by a follow-up procedure conducted by gynaecologists after identification of an expected benign cyst in the ovaries and the woman may return to her GP with the message that the gynaecologist found nothing suspicious. Jessen et al. demonstrated a later increase in the use of CT scans, abdominal ultrasound, and CPP referrals than found in the present study [11]. Differences in the definitions of reference group and outcomes may explain the discrepancy. For example, the study by Jessen et al. included only referrals to abdominal CPPs [11]. We demonstrated a notably lower relative use of CT scans among cases compared to controls until six months prior to diagnosis. However, the absolute number of examinations was small.

The studies by Hansen et al. [10] and Jessen et al. [11] suggest that some abdominal cancers could be detected at an earlier time point, revealing the existence of a “diagnostic time window”. However, compared to references, increased healthcare use in general practice was seen closely to the time of the diagnosis in OC patients. OC has been defined as a hard-to-suspect cancer [29], suggesting that also patient’s delay is an important aspect in OC. This is supported by Seibaek et al., who found that the delay in OC diagnosis also depended on the women’s interpretation of their symptoms, which was influenced by their personal experiences and by their cultural and social background [30]. Furthermore, normalisation of bodily changes and interpretation of symptoms as consequences of diet, age, hormonal imbalance, or being female have also been suggested, and vague symptoms have been reported as a barrier for help-seeking [31]. Although the results of this present study indicated a narrow window with increased activity in primary care, the conversation between the woman and her GP may have changed to relate to symptoms arising from the lower body long before diagnosis, but without prompting higher frequency of encounters to the GP. Thus, our findings underline the pivotal role of safety-netting as well as the importance of OC awareness when GPs see patients with vague unspecific symptoms, who may have low risk, but not no risk of malignant disease [32, 33].

## Conclusion

Increased use of both primary and secondary care was seen shortly before an OC diagnosis. This indicates a narrow window of opportunity for a timelier diagnosis. However, increased use of TVUS and gynaecologist was seen from six to eight months before diagnosis, which could suggest the presence of early signs of OC. GP awareness of ovarian malignancies and safety-netting may be pivotal in consultations with women presenting with unspecific symptoms of potential OC, who may be at low risk, but not no risk of malignant disease.

### Electronic supplementary material

Below is the link to the electronic supplementary material.

**Supplementary Material 1: Appendix A**. Overview of procedure codes used to define outcomes of the paper

**Supplementary Material 2: Appendix B**. Monthly incidence rate ratios (IRR) including IRRs in the last month prior to an ovarian cancer or borderline ovarian tumour diagnosis

**Supplementary Material 3: Appendix C**. Incidence rate ratios with 95% confidence intervals for all outcomes performed in the 1-12 months before an ovarian cancer diagnosis compared to the reference group

## Data Availability

In accordance with Danish regulations, all data was stored at Statistics Denmark on secure servers. Hence, the data that support the findings of this study are not publicly available in accordance with the Danish regulations of research. The corresponding author can be contacted for further information.

## List of abbreviations

BOT: Borderline ovarian tumour
CCI: Charlson Comorbidity Index
CI: Confidence interval
CPP: Cancer patient pathway
CT: Computed tomography
DCR: Danish Cancer Registry
DGCD: Danish Gynecological Cancer Database
DNPR: Danish National Patient Register
FIGO: International Federation of Gynecology and Obstetrics
GP: General practitioner
Hb: Haemoglobin
ICD-10: International Classification of Diseases, 10th revision
IQR: Interquartile range
IRR: Incidence rate ratio
OC: Ovarian cancer
POCT: Point-of-care test
RR: Risk ratio
TVUS: Transvaginal ultrasound
