# Supplementary material for: Healthcare use and clinical investigations before a diagnosis of ovarian cancer: a register-based study in Denmark
Source: BMC Prim Care. 2023 Aug 30;24:169. doi: 10.1186/s12875-023-02132-3 (PMC10466681; doi:10.1186/s12875-023-02132-3)
Supplement: Supplementary file 1 — Supplementary Material 1: Appendix A. Overview of procedure codes used to define outcomes of the paper [file 12875_2023_2132_MOESM1_ESM.docx]

**Appendix A:**

**Overview of procedure codes used to define outcomes of the paper**

Data on procedure codes were obtained from the Danish National Patient Register (DNPR) and the Danish National Health Insurance Service Register (NHSR).

DNPR: information on consultations and investigations performed at both public (DNPR) and private hospitals (DNPR private), and Cancer Patient Pathways (CPPs) performed at public hospitals.

NHSR: information on consultations and investigations performed by private specialists, including general practitioners (GPs).
We included procedure codes in 2012-2018 from general practice (speciality 80), diagnostic radiology (05), gynaecology (07), internal medicine (08) and surgery (09).

| **Procedure codes for consultations and services by general practitioners**  **(Speciality 80)** |
| --- |
| **NHSR** |
| **0101 –** Face-to-face consultation, daytime |
| **0105 –** Consultation by e-mail |
| **0201 –** Consultation by telephone, daytime |
| **0411 –** Home visit (less than 4 km from the clinic) |
| **0421 –** Home visit (5 to 8 km from the clinic) |
| **0431 –** Home visit (9 to 12 km from the clinic) |
| **0441 –** Home visit (13 to 16 km from the clinic) |
| **0451** – Home visit (17 to 20 km from the clinic) |
| **0461** – Home visit (21 km or more from the clinic) |
| **0491 –** Home visit (on the way) |
| **2101 –** Blood test |
| **7101 –** Urine dipstick test |
| **7108** – Haemoglobin measurement (point-of-care testing) |

| **Procedure codes for colonoscopies, including sigmoidoscopy** | |
| --- | --- |
| **NHSR** | **DNPR public and DNPR private** |
| **Speciality 8: 2307** - Sigmoidoscopy | **KUJF32** - Colonoscopy |
| **Speciality 8: 2308** - Colonoscopy | **KUJF35** – Colonoscopy with biopsy |
| **Speciality 9: 2114** - Colonoscopy | **KUJF42** - Sigmoidoscopy |
| **Speciality 9: 2307** - Sigmoidoscopy | **KUJF45** - Sigmoidoscopy with biopsy |

| **Procedure codes for gastroscopies** | |
| --- | --- |
| **NHSR** | **DNPR public and DNPR private** |
| **Speciality 8: 2302** - Gastroscopy | **KUJC02** – Esophagoscopy |
| **Speciality 9: 2302** - Gastroscopy | **KUJC05** – Esophagoscopy with biopsy |
|  | **KUJC12** – Flexible esophagoscopy |
|  | **KUJC15** – Flexible esophagoscopy with biopsy |
|  | **KUJD02** – Gastroscopy |
|  | **KUJD05** – Gastroscopy with biopsy |

| **Procedure codes for transvaginal ultrasound (TVUS)** | |
| --- | --- |
| **NHSR** | **DNPR public and DNPR private** |
| **Speciality 7*:* 0110** - First consultation, including TVUS | **UXUD82** - TVUS of genitalia feminina |
| **Speciality 7*:* 0130** – Follow-up consultation, including TVUS | **UXUD82A** - TVUS of uterus with contrast |
| **Speciality 7*:* 2112** - Ultrasound | **UXUD82B** – TVUS of uterus and salpinges with contrast |

| **Procedure codes for abdominal ultrasound** | |
| --- | --- |
| **NHSR** | **DNPR public and DNPR private** |
| **Speciality 5*:* 2160** – Ultrasound of the upper abdomen | **UXUD** - General ultrasound of the abdomen |
| **Speciality 5*:* 2162** – Ultrasound of the lower abdomen | **UXUD10** - Ultrasound of the upper abdomen |
| **Speciality 5*:* 2163** – Ultrasound of the lower abdomen with focus on the bladder | **UXUD12** – Ultrasound of the intestines |
| **Speciality 5: 2151** – Ultrasound of the kidney and the urinary system | **UXUD15** – Ultrasound of the lower abdomen UL |
| **Speciality 8: 2309** – Ultrasound of the upper abdomen | **UXUD20** – Ultrasound of the retroperitoneum |
| **Speciality 9: 5009** – Ultrasound of the upper abdomen | **UXUD22** – Ultrasound of the abdominal wall |
|  | **UXUD61** – Ultrasound of the kidneys |
|  | **UXUD70** – Ultrasound of the liver |
|  | **UXUD75** – Ultrasound of the bladder |
|  | **UXUD80** – Ultrasound of the genitalia feminina |
|  | **UXUD85** – Ultrasound of the cervix |

| **Procedure codes for abdominal CT** | |
| --- | --- |
| **NHSR** | **DNPR public and DNPR private** |
| **Speciality 5: 5019** – CT urography | **UXCD** - CT abdomen and pelvis |
| **Speciality 5*:* 5020** - CT urinary tract system | **UXCD00** – CT abdomen |
|  | **UXCD10** – CT upper abdomen |
|  | **UXCD15** – CT lower abdomen, including pelvis |
|  | **UXCD20** – CT retroperitoneum |
|  | **UXCD40** – CT liver |
|  | **UXCD55** – CT pancreas |
|  | **UXCD60** – CT kidney |
|  | **UXCD61** – CT kidney and urinary tract system |
|  | **UXCD62** - CT urography |
|  | **UXCD65** - CT adrenal glands |
|  | **UXCD75** – CT bladder |
|  | **UXCD80** – CT colonography |

| **Procedure codes for abdominal MRI** | |
| --- | --- |
| **NHSR** | **DNPR public and DNPR private** |
| Abdominal/pelvic MRIs are not performed by private specialists | **UXMD** – MRI abdomen and pelvis |
|  | **UXMD10** – MRI upper abdomen |
|  | **UXMD15** – MRI lower abdomen, including pelvis |
|  | **UXMD20** – MRI retroperitoneum |
|  | **UXMD22** – MRI abdominal wall |
|  | **UXMD25** – MRI stomach and gastrointestinal system |
|  | **UXMD25A** – MRI small intestine |
|  | **UXMD25B** – MRI large intestine |
|  | **UXMD40** - MRI liver |
|  | **UXMD50** – MR cholangio­pancreatography (MRCP) |
|  | **UXMD55** – MRI pancreas |
|  | **UXMD60** – MRI kidney |
|  | **UXMD62** – MRI urography |
|  | **UXMD65** – MRI adrenal glands |
|  | **UXMD75** – MRI bladder |
|  | **UXMD80** – MRI genitalia feminina |
|  | **UXMH00** – Whole body MRI |

| **Procedure codes for cancer patient pathways (CPPs)** |
| --- |
| **DNPR public**  (CPP’s are only performed at public hospitals) |
| **AFBxxA** – referral to CPP, start: |
| **01** Breast cancer |
| **02** Head and neck cancer |
| **03** Lymphomas and chronic lymphocytic leukaemia |
| **04** Myelomatosis |
| **05** Acute leukaemia and advanced myelodysplastic syndrome |
| **06** Chronic myeloid disease |
| **07** Pancreatic cancer |
| **08** Biliary tract cancer |
| **09** Oesophageal/gastric cancer |
| **10** Liver cancer |
| **11** Colorectal metastases in the liver |
| **12** Colorectal cancer |
| **13** Urinary tract cancer |
| **14** Bladder cancer |
| **15** Kidney cancer |
| **19** Cancer in exterior female genitalia |
| **20** Endometrial cancer |
| **22** Cervix cancer |
| **23** Brain cancer |
| **24** Eye and orbital cancer |
| **25** Malignant melanoma of skin |
| **26** Lung cancer |
| **27** Bone sarcomas |
| **29** Sarcomas in non-bone tissue |
| **31** Anal cancer |
| **32** Cancer of the renal pelvis or ureter |
| **33** Pleural cancer |
| **AFA01A** – Serious non-specific symptoms and signs of cancer, referral, start |
