# Supplementary material for: Healthcare use and clinical investigations before a diagnosis of ovarian cancer: a register-based study in Denmark
Source: BMC Prim Care. 2023 Aug 30;24:169. doi: 10.1186/s12875-023-02132-3 (PMC10466681; doi:10.1186/s12875-023-02132-3)
Supplement: Supplementary file 2 — Supplementary Material 2: Appendix B. Monthly incidence rate ratios (IRR) including IRRs in the last month prior to an ovarian cancer or borderline ovarian tumour diagnosis [file 12875_2023_2132_MOESM2_ESM.docx]

**Appendix B: Monthly incidence rate ratios (IRR) including IRRs in the last month prior to an ovarian cancer or borderline ovarian tumour diagnosis**

**Figure S1 –** Consultation rates and tests in general practice in the year preceding an ovarian cancer diagnosis

Urine dipstick tests

GP visits (face-to-face)


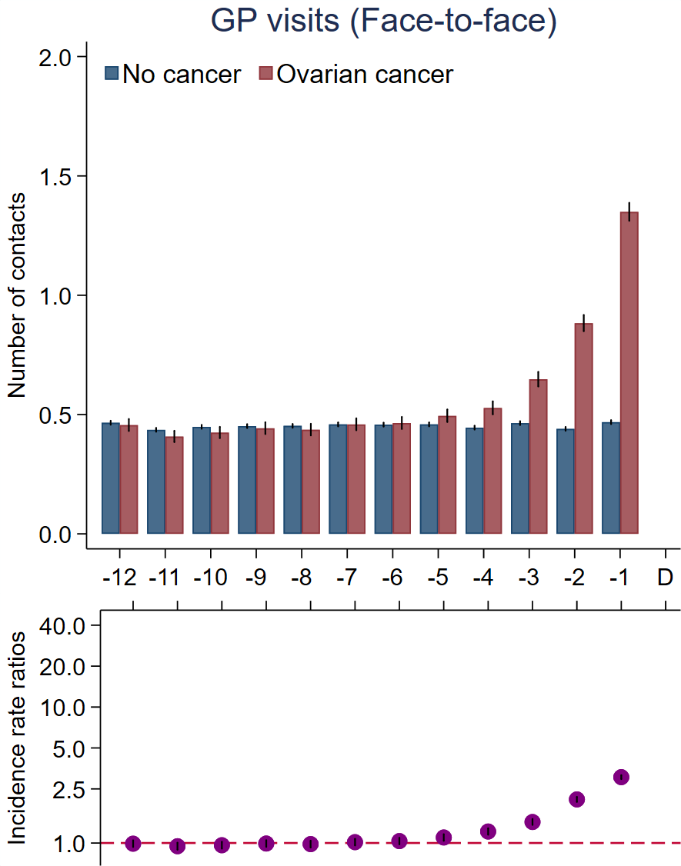

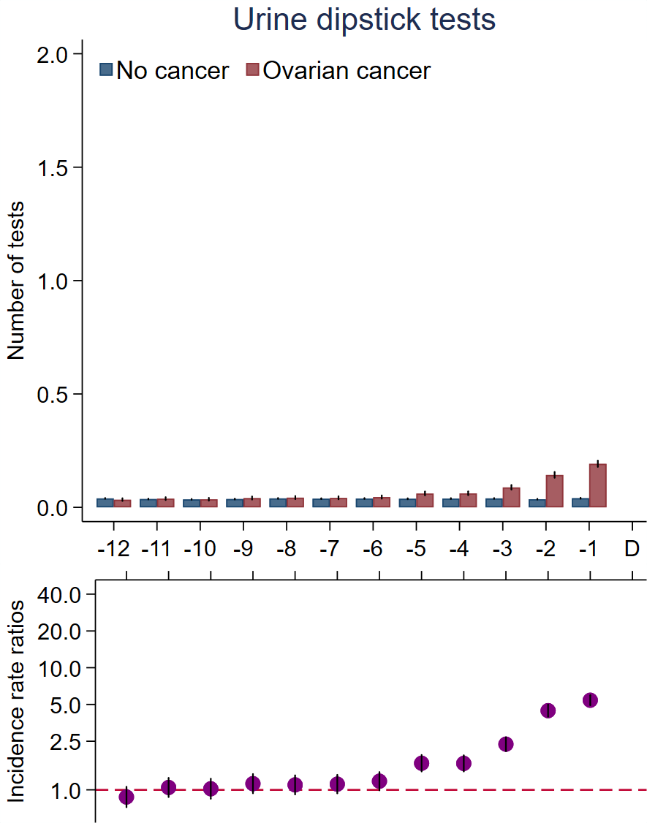


*b*

*a*

Blood tests

Haemoglobin measurements


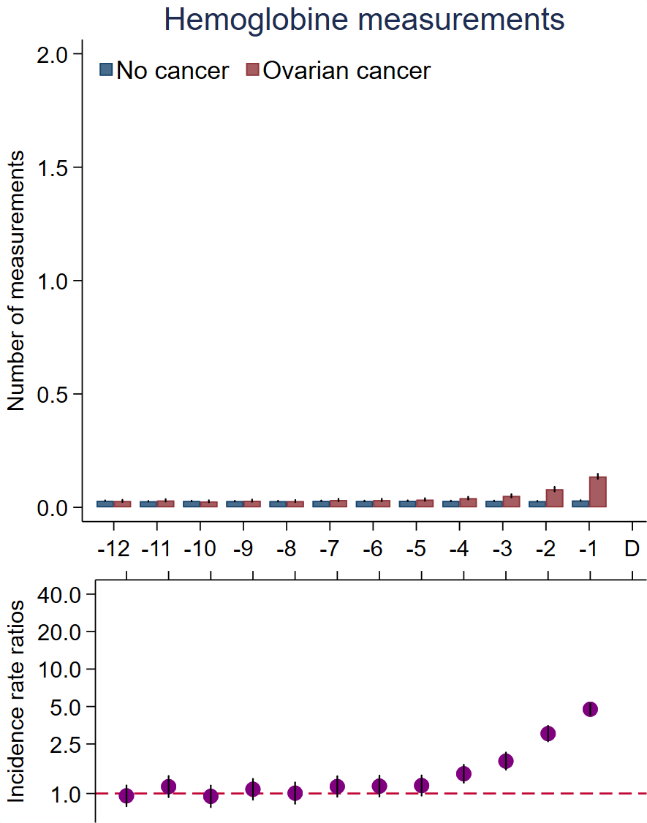

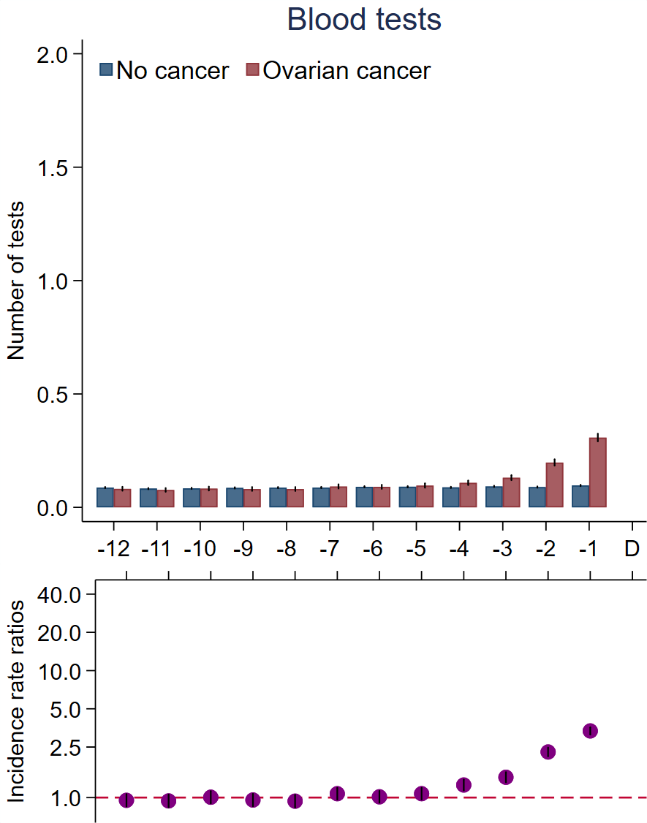


*d*

*c*

Number of contacts or tests in general practice stratified on ovarian cancer (yes/no). Borderline ovarian tumours included. Number of contacts/tests are presented as crude rates of mean number of contacts/tests per month. Incidence rate ratios were adjusted for age, comorbidity, educational level, marital status, disposable income and country of origin. Black lines represent 95% confidence intervals.

**Figure S2 –** Contacts to relevant specialists in the year preceding an ovarian cancer diagnosis

Department of gynaecology

Private practicing gynaecologists


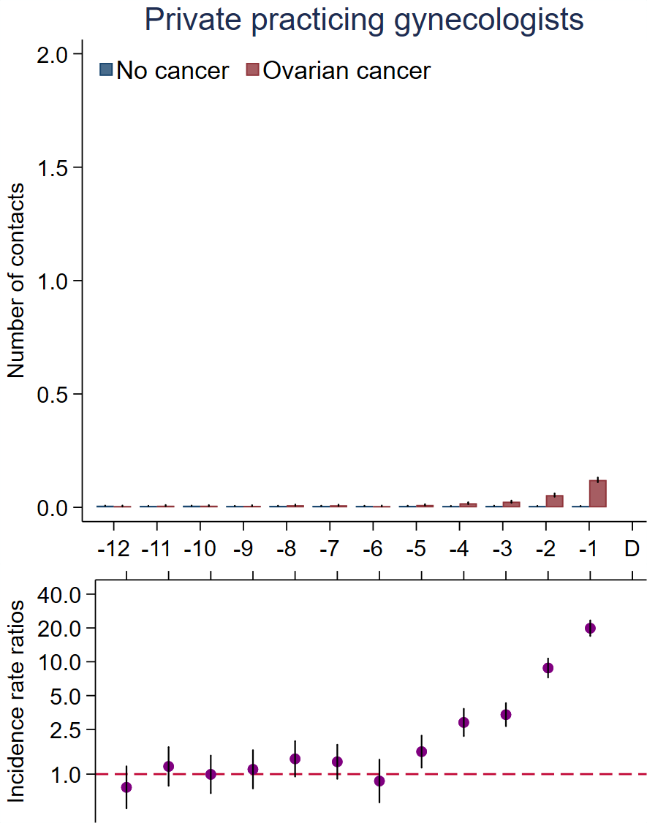

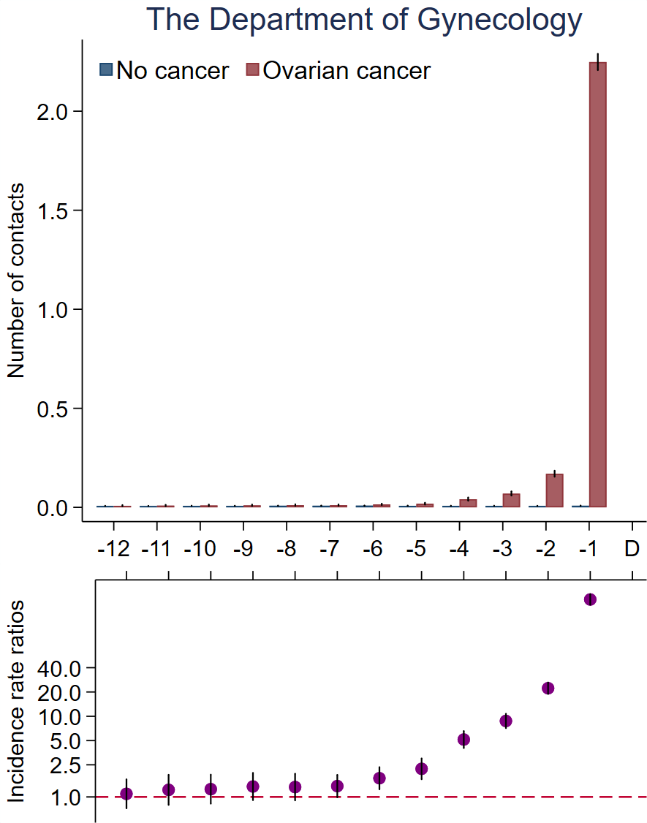


*a*

*b*

*c*

Department of urology


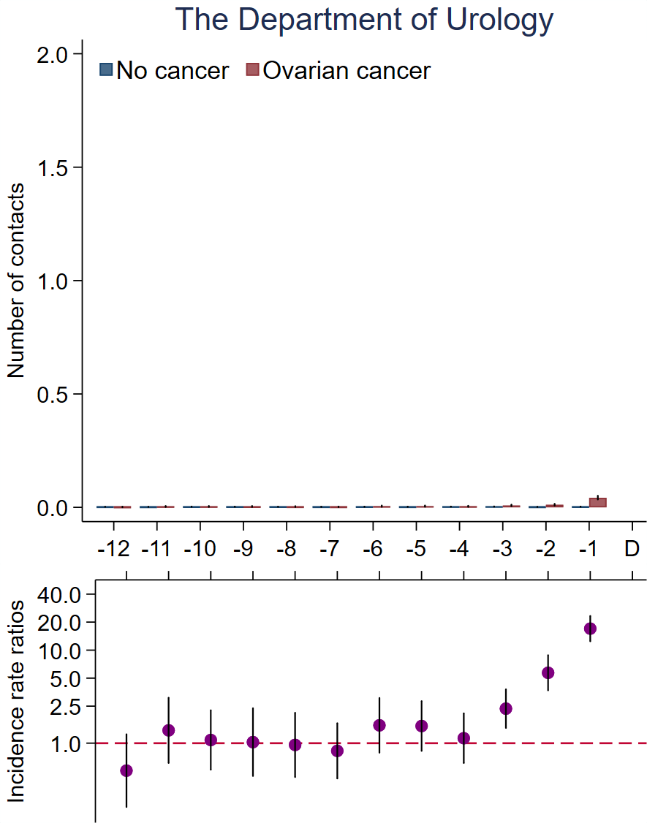


Number of consultations in different healthcare departments stratified on ovarian cancer (yes/no). Borderline ovarian tumours included. Maximum one contact at each department/private specialist per women per day included. Number of consultations are presented as crude rates of mean number of consultations per month. Incidence rate ratios were adjusted for age, comorbidity, educational level, marital status, disposable income and country of origin. Black lines represent 95% confidence intervals.

**Figure S3 –** Diagnostic investigations made by private and hospital specialists in the year preceding an ovarian cancer diagnosis

Colonoscopies

Transvaginal ultrasound (TVUS)


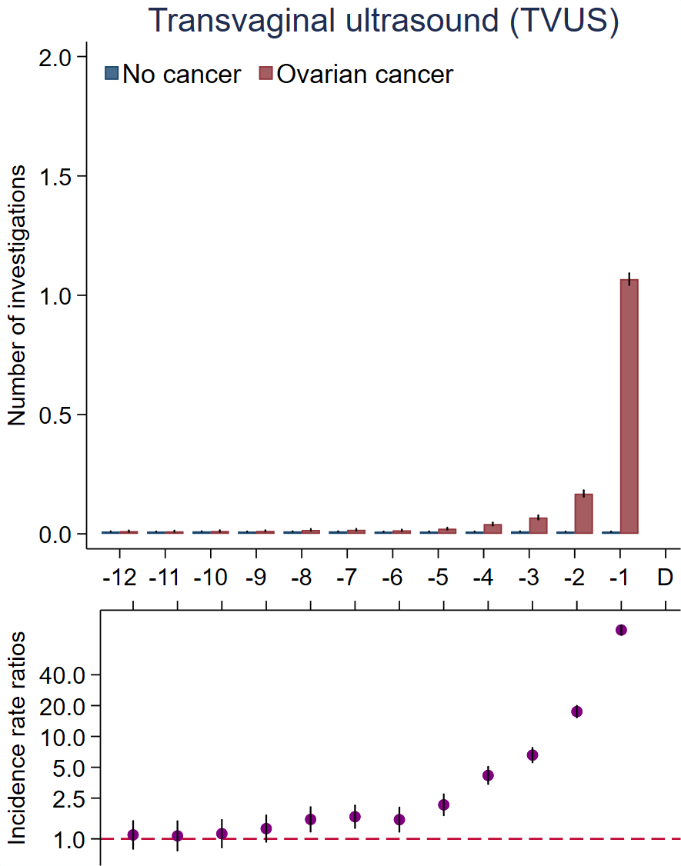

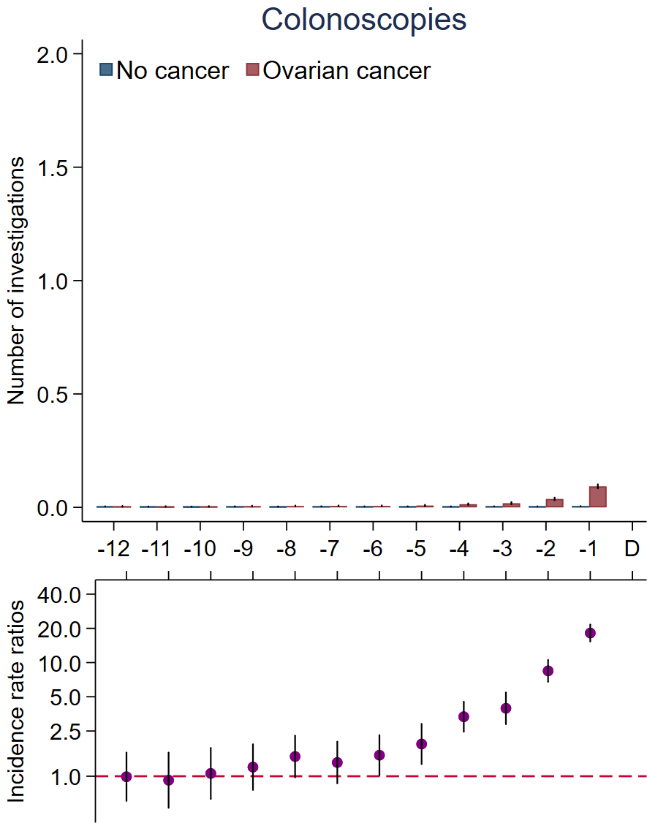


*a*

*b*

Computed tomography (CT)

Abdominal ultrasound


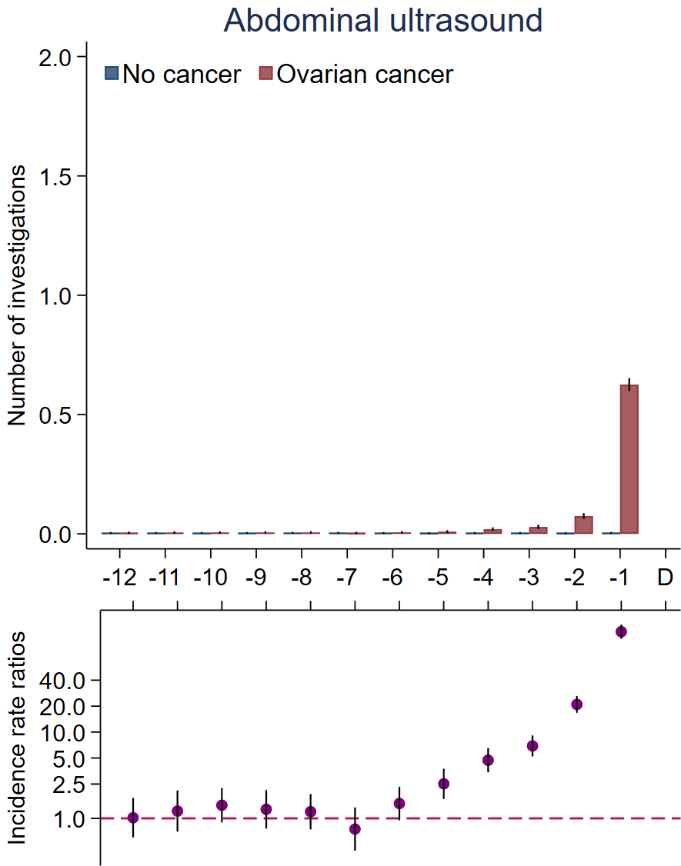

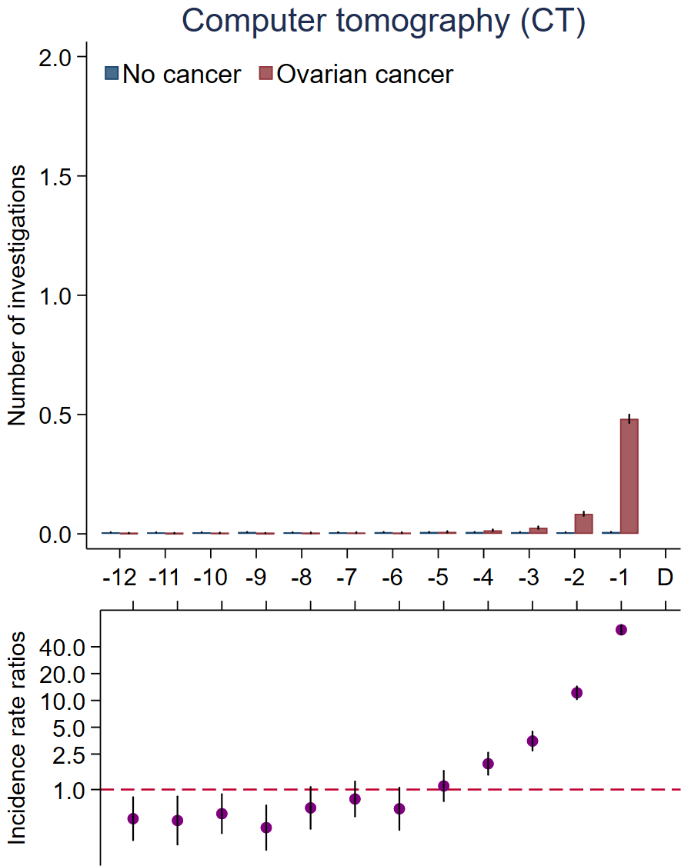


*c*

*d*

Number of different diagnostic investigations performed stratified on ovarian cancer (yes/no). Borderline ovarian tumours included. Maximum one investigation of each type per women per day included. Number of investigations are presented as crude rates of mean number of investigations per month. Incidence rate ratios were adjusted for age, comorbidity, educational level, marital status, disposable income and country of origin. Black lines represent 95% confidence intervals.
CT scans were only performed at hospitals.

**Figure S4 –** Cancer patient pathway referrals in the year preceding an ovarian cancer diagnosis. The CPP for OC was not included.

CPP referrals (CPP for OC not included)


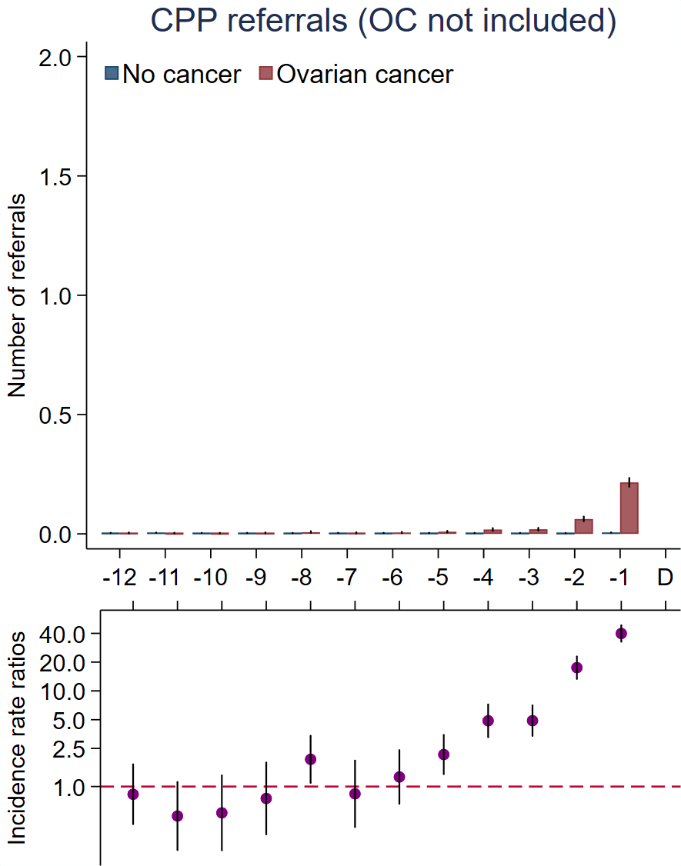


Number of CPP referrals from 2014-2018 stratified on ovarian cancer (yes/no). Borderline ovarian tumours included. Maximum one CPP referral per women per day included. CPP for ovarian cancer not included. Number of investigations are presented as crude rates of mean number of CPPs per month. Incidence rate ratios were adjusted for age, comorbidity, educational level, marital status, disposable income and country of origin. Black lines represent 95% confidence intervals.

Abbreviations: CPP = cancer patient pathway; OC = ovarian cancer
