# Supplementary material for: Healthcare use and clinical investigations before a diagnosis of ovarian cancer: a register-based study in Denmark
Source: BMC Prim Care. 2023 Aug 30;24:169. doi: 10.1186/s12875-023-02132-3 (PMC10466681; doi:10.1186/s12875-023-02132-3)
Supplement: Supplementary file 3 — Supplementary Material 3: Appendix C. Incidence rate ratios with 95% confidence intervals for all outcomes performed in the 1-12 months before an ovarian cancer diagnosis compared to the reference group [file 12875_2023_2132_MOESM3_ESM.docx]

**Appendix C:**

**Incidence rate ratios with 95% confidence intervals for all outcomes performed in the 1-12 months before an ovarian cancer diagnosis compared to the reference group**

**Table S1.** Incidence rate ratios (IRR) with 95% confidence intervals for face-to-face consultations (F2F), haemoglobin measurements (Hb) (point-of-care test), blood tests and urine dipstick tests (U-tests) performed in general practice in the 1-12 months before an ovarian cancer or borderline ovarian tumour diagnosis compared to the reference group.

|  | **-12** | | **-11** | | **-10** | | **-9** | | **-8** | | **-7** | |
| --- | --- | --- | --- | --- | --- | --- | --- | --- | --- | --- | --- | --- |
|  | **IRR** | **95% CI*** | **IRR** | **95% CI*** | **IRR** | **95% CI*** | **IRR** | **95% CI*** | **IRR** | **95% CI*** | **IRR** | **95% CI*** |
| **F2F** | 0.99 | 0.93-1.05 | 0.95 | 0.89-1.01 | 0.96 | 0.91-1.02 | 0.99 | 0.94-1.05 | 0.98 | 0.93-1.04 | 1.02 | 0.96-1.08 |
| **Hb** | 0.96 | 0.79-1.16 | 1.14 | 0.93-1.38 | 0.95 | 0.78-1.16 | 1.08 | 0.89-1.31 | 1.01 | 0.83-1.23 | 1.14 | 0.94-1.38 |
| **Blood tests** | 0.95 | 0.85-1.07 | 0.94 | 0.84-1.06 | 1.01 | 0.90-1.13 | 0.96 | 0.85-1.07 | 0.94 | 0.84-1.05 | 1.07 | 0.96-1.20 |
| **U-tests** | 0.87 | 0.72-1.06 | 1.05 | 0.87-1.25 | 1.02 | 0.84-1.23 | 1.12 | 0.94-1.35 | 1.10 | 0.92-1.32 | 1.11 | 0.94-1.33 |
|  | **-6** | | **-5** | | **-4** | | **-3** | | **-2** | | **-1** | |
|  | **IRR** | **95% CI*** | **IRR** | **95% CI*** | **IRR** | **95% CI*** | **IRR** | **95% CI*** | **IRR** | **95% CI*** | **IRR** | **95% CI*** |
| **F2F** | 1.03 | 0.98-1.10 | **1.10** | **1.04-1.16** | **1.22** | **1.15-1.29** | **1.43** | **1.36-1.50** | **2.09** | **2.00-2.19** | **3.05** | **2.95-3.16** |
| **Hb** | 1.15 | 0.94-1.40 | 1.16 | 0.96-1.40 | **1.44** | **1.22-1.71** | **1.82** | **1.55-2.14** | **3.03** | **2.62-3.50** | **4.76** | **4.28-5.30** |
| **Blood tests** | 1.02 | 0.91-1.13 | 1.07 | 0.97-1.20 | **1.26** | **1.14-1.39** | **1.45** | **1.32-1.59** | **2.29** | **2.11-2.48** | **3.35** | **3.13-3.58** |
| **U-tests** | 1.18 | 0.99-1.40 | **1.65** | **1.41-1.93** | **1.65** | **1.41-1.92** | **2.37** | **2.07-2.70** | **4.45** | **3.97-5.00** | **5.42** | **4.92-5.97** |

*IRRs adjusted for age, disposable income, educational level, marital status, country of origin and comorbidity according to the Charlson Comorbidity Index. Significant findings in bold.

**Table S2.** Incidence rate ratios (IRR) with 95% confidence intervals for colonoscopies, abdominal ultrasound (US), transvaginal ultrasound (TVUS) and computed tomography (CT) scans performed at hospital and by private practicing medical specialists in the 1-12 months before an ovarian cancer or borderline ovarian tumour diagnosis compared to the reference group.

|  | **-12** | | **-11** | | **-10** | | **-9** | | | **-8** | | **-7** | |
| --- | --- | --- | --- | --- | --- | --- | --- | --- | --- | --- | --- | --- | --- |
|  | **IRR** | **95% CI*** | **IRR** | **95% CI*** | **IRR** | **95% CI*** | **IRR** | **95% CI*** | | **IRR** | **95% CI*** | **IRR** | **95% CI*** |
| **Colonoscopies** | 0.99 | 0.61-1.62 | 0.92 | 0.53-1.62 | 1.06 | 0.63-1.78 | 1.20 | 0.76-1.91 | | 1.49 | 0.98-2.28 | 1.32 | 0.87-2.02 |
| **Abdominal US** | 1.02 | 0.61-1.70 | 1.22 | 0.72-2.07 | 1.42 | 0.92-2.20 | 1.27 | 0.77-2.10 | | 1.20 | 0.76-1.88 | 0.75 | 0.43-1.32 |
| **TVUS** | 1.09 | 0.80-1.50 | 1.07 | 0.77-1.49 | 1.12 | 0.82-1.53 | 1.26 | 0.93-1.70 | | **1.55** | **1.17-2.04** | **1.65** | **1.28-2.12** |
| **CT** | **0.47** | **0.27-0.82** | **0.45** | **0.24-0.84** | **0.54** | **0.32-0.89** | **0.37** | **0.21-0.67** | | 0.62 | 0.36-1.07 | 0.78 | 0.50-1.23 |
|  | **-6** | | **-5** | | **-4** | | **-3** | | | **-2** | | **-1** | |
|  | **IRR** | **95% CI*** | **IRR** | **95% CI*** | **IRR** | **95% CI*** | **IRR** | **95% CI*** | **IRR** | | **95% CI*** | **IRR** | **95% CI*** |
| **Colonoscopies** | **1.53** | **1.02-2.30** | **1.92** | **1.28-2.89** | **3.34** | **2.46-4.53** | **3.96** | **2.87-5.47** | **8.46** | | **6.76-10.58** | **18.21** | **15.32-21.63** |
| **Abdominal US** | 1.49 | 0.97-2.29 | **2.52** | **1.71-3.70** | **4.72** | **3.48-6.40** | **6.92** | **5.33-8.99** | **20.95** | | **17.02-25.78** | **146.00** | **122.91-173.44** |
| **TVUS** | **1.54** | **1.17-2.03** | **2.15** | **1.70-2.73** | **4.17** | **3.42-5.07** | **6.58** | **5.58-7.76** | **17.49** | | **15.30-19.98** | **109.44** | **98.18-121.99** |
| **CT** | 0.61 | 0.35-1.05 | 1.10 | 0.74-1.63 | **1.95** | **1.46-2.60** | **3.50** | **2.73-4.49** | **12.18** | | **10.30-14.42** | **62.04** | **55.07-69.89** |

*IRRs adjusted for age, disposable income, educational level, marital status, country of origin and comorbidity according to the Charlson Comorbidity Index. Significant findings in bold.

**Table S3.** Incidence rate ratios (IRR) with 95% confidence intervals for contacts to a department of urology (Dep. Uro.), a department of gynaecology (Dep. Gyn.) and private practicing gynaecologist (Priv. Gyn.) in the 1-12 months before an ovarian cancer or borderline ovarian tumour diagnosis compared to the reference group.

|  | **-12** | | **-11** | | **-10** | | **-9** | | **-8** | | **-7** | |
| --- | --- | --- | --- | --- | --- | --- | --- | --- | --- | --- | --- | --- |
|  | **IRR** | **95% CI*** | **IRR** | **95% CI*** | **IRR** | **95% CI*** | **IRR** | **95% CI*** | **IRR** | **95% CI*** | **IRR** | **95% CI*** |
| **Dep. Uro.** | 0.51 | 0.21-1.25 | 1.37 | 0.61-3.09 | 1.08 | 0.52-2.26 | 1.03 | 0.44-2.38 | 0.96 | 0.43-2.13 | 0.83 | 0.42-1.64 |
| **Dep. Gyn.** | 1.09 | 0.72-1.66 | 1.22 | 0.79-1.89 | 1.25 | 0.82-1.89 | 1.35 | 0.91-1.99 | 1.33 | 0.91-1.94 | 1.36 | 0.99-1.88 |
| **Priv. Gyn.** | 0.76 | 0.50-1.18 | 1.17 | 0.79-1.74 | 1.00 | 0.68-1.46 | 1.10 | 0.74-1.64 | 1.37 | 0.95-1.97 | 1.29 | 0.91-1.83 |
|  | **-6** | | **-5** | | **-4** | | **-3** | | **-2** | | **-1** | |
|  | **IRR** | **95% CI*** | **IRR** | **95% CI*** | **IRR** | **95% CI*** | **IRR** | **95% CI*** | **IRR** | **95% CI*** | **IRR** | **95% CI*** |
| **Dep. Uro.** | 1.56 | 0.79-3.07 | 1.53 | 0.83-2.84 | 1.13 | 0.61-2.10 | **2.35** | **1.46-3.80** | **5.72** | **3.69-8.84** | **17.05** | **12.45-23.35** |
| **Dep. Gyn.** | **1.71** | **1.24-2.35** | **2.23** | **1.66-3.01** | **5.15** | **4.03-6.58** | **8.75** | **7.12-10.77** | **22.29** | **18.87-26.31** | **281.00** | **241.46-327.03** |
| **Priv. Gyn.** | 0.87 | 0.56-1.35 | **1.59** | **1.14-2.20** | **2.89** | **2.18-3.82** | **3.38** | **2.67-4.29** | **8.81** | **7.25-10.71** | **19.91** | **16.95-23.38** |

*IRRs adjusted for age, disposable income, educational level, marital status, country of origin and comorbidity according to the Charlson Comorbidity Index. Significant findings in bold.

**Table S4.** Incidence rate ratios (IRR) with 95% confidence intervals for cancer patient pathway (CPP) referrals (omitting CPP for ovarian cancer) in the 1-12 months before an ovarian cancer or borderline ovarian tumour diagnosis compared to the reference group. Omitting women diagnosed between 2012-2014 (see main text).

|  | **-12** | | **-11** | | **-10** | | **-9** | | **-8** | | **-7** | |
| --- | --- | --- | --- | --- | --- | --- | --- | --- | --- | --- | --- | --- |
|  | **IRR** | **95% CI*** | **IRR** | **95% CI*** | **IRR** | **95% CI*** | **IRR** | **95% CI*** | **IRR** | **95% CI*** | **IRR** | **95% CI*** |
| **CPP** | 0.83 | 0.40-1.71 | 0.49 | 0.22-1.12 | 0.53 | 0.22-1.31 | 0.75 | 0.32-1.79 | **1.93** | **1.09-3.40** | 0.84 | 0.38-1.87 |
|  | **-6** | | **-5** | | **-4** | | **-3** | | **-2** | | **-1** | |
|  | **IRR** | **95% CI*** | **IRR** | **95% CI*** | **IRR** | **95% CI*** | **IRR** | **95% CI*** | **IRR** | **95% CI*** | **IRR** | **95% CI*** |
| **CPP** | 1.26 | 0.66-2.41 | **2.16** | **1.35-3.47** | **4.88** | **3.28-7.24** | **4.89** | **3.38-7.08** | **17.5** | **13.34-22.99** | **39.87** | **32.66-48.68** |

*IRRs adjusted for age, disposable income, educational level, marital status, country of origin and comorbidity according to the Charlson Comorbidity Index. Significant findings in bold.
